# In Memoriam: Prof. Dr. José Antônio Livramento

**DOI:** 10.1055/s-0045-1813244

**Published:** 2025-12-08

**Authors:** Luís dos Ramos Machado

**Affiliations:** 1 Laboratório de Neurodiagnóstico Spina França, São Paulo SP, Brazil.

**Figure 1:**
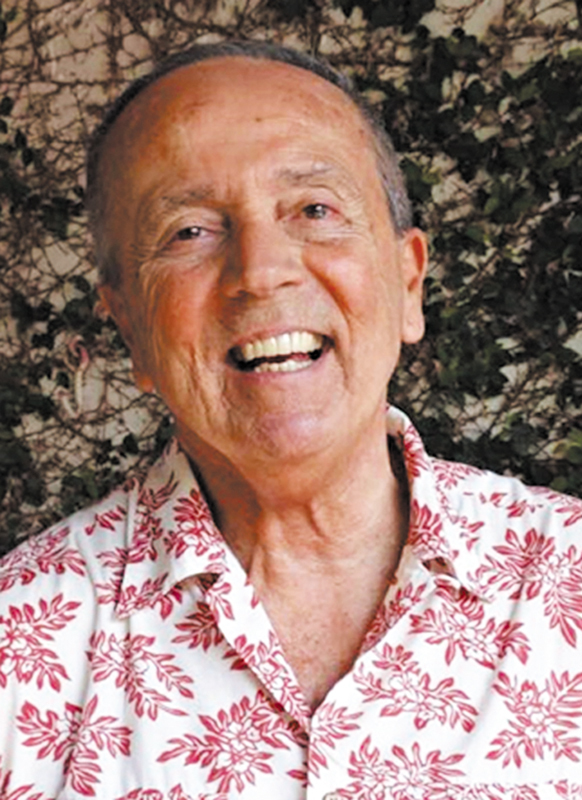
Professor Dr. José Antônio Livramento

A person with a joyful and relaxed spirit, a loyal lover of the music of Caetano Veloso, Maria Bethânia, and Gal Costa. He loved to host gatherings at his home with friends, in the best style of weekends at the beaches of his beloved city of Recife.

Due to a family issue, shortly after graduation, he had to abandon the project of an internship in France and came to São Paulo. Here, he forged an instant friendship with Prof. Spina França, who immediately considered him as another son in the family.

From the beginning, his focus was on diagnostic neurology, to which he dedicated himself with commitment and competence throughout his career. Two areas of work, parallel and complementary: the Center for Neurology Investigations, currently the Medical Research Laboratory of the Faculty of Medicine at Universidade de São Paulo (LIM 15), and, outside the university, the Spina França Neurodiagnostic Laboratory.

He dedicated himself to the research lines of Prof. Spina's school of tropical neurology and, later, to the methodological standardization of markers for Alzheimer's disease. In the laboratory, he was a joyful and kind person, emphasizing encouragement and support for residents and interns, especially during their learning stage.

He served as the General Secretary of the Brazilian Academy of Neurology (Academia Brasileira de Neurologia, in Portuguese) for 4 years, with an impeccable administration. In addition, he held the position of Co-Editor-in-Chief of the journal *Arquivos de Neuro-Psiquiatria* for 6 years. Through diligent and dedicated work, the journal's impact factor significantly increased during this time.

There is sadness at the loss of a friendly, pleasant, competent, and hardworking individual.

